# Supplementary material for: Amorphous nickel-cobalt complexes hybridized with 1T-phase molybdenum disulfide via hydrazine-induced phase transformation for water splitting
Source: Nat Commun. 2017 May 9;8:15377. doi: 10.1038/ncomms15377 (PMC5436140; doi:10.1038/ncomms15377)
Supplement: Supplementary Information — Supplementary Figures and Supplementary Tables [file ncomms15377-s1.pdf]

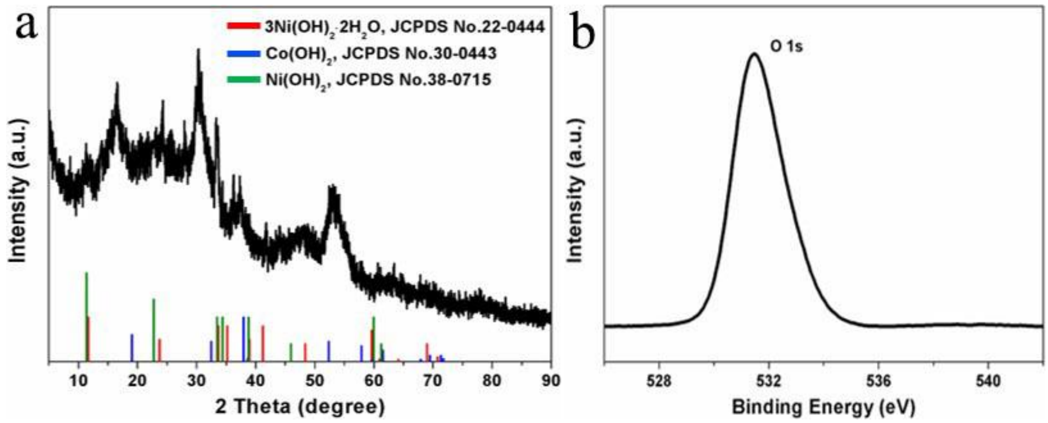


**Supplementary Figure 1.** (a) XRD pattern of NCUNs. The red lines present the standard nickel hydroxide hydrate (JCPDS No. 22-0444) peaks, the blue lines demonstrate the standard cobalt hydroxide (JCPDS No. 30-0443) peaks and the olive lines display the standard nickel hydroxide (JCPDS No. 38-0715) peaks. (b) XPS spectrum of O 1s orbital in NCUNs. The peak was located at 531.5 eV, which was the position of characteristic peak of O 1s orbital in hydroxides.

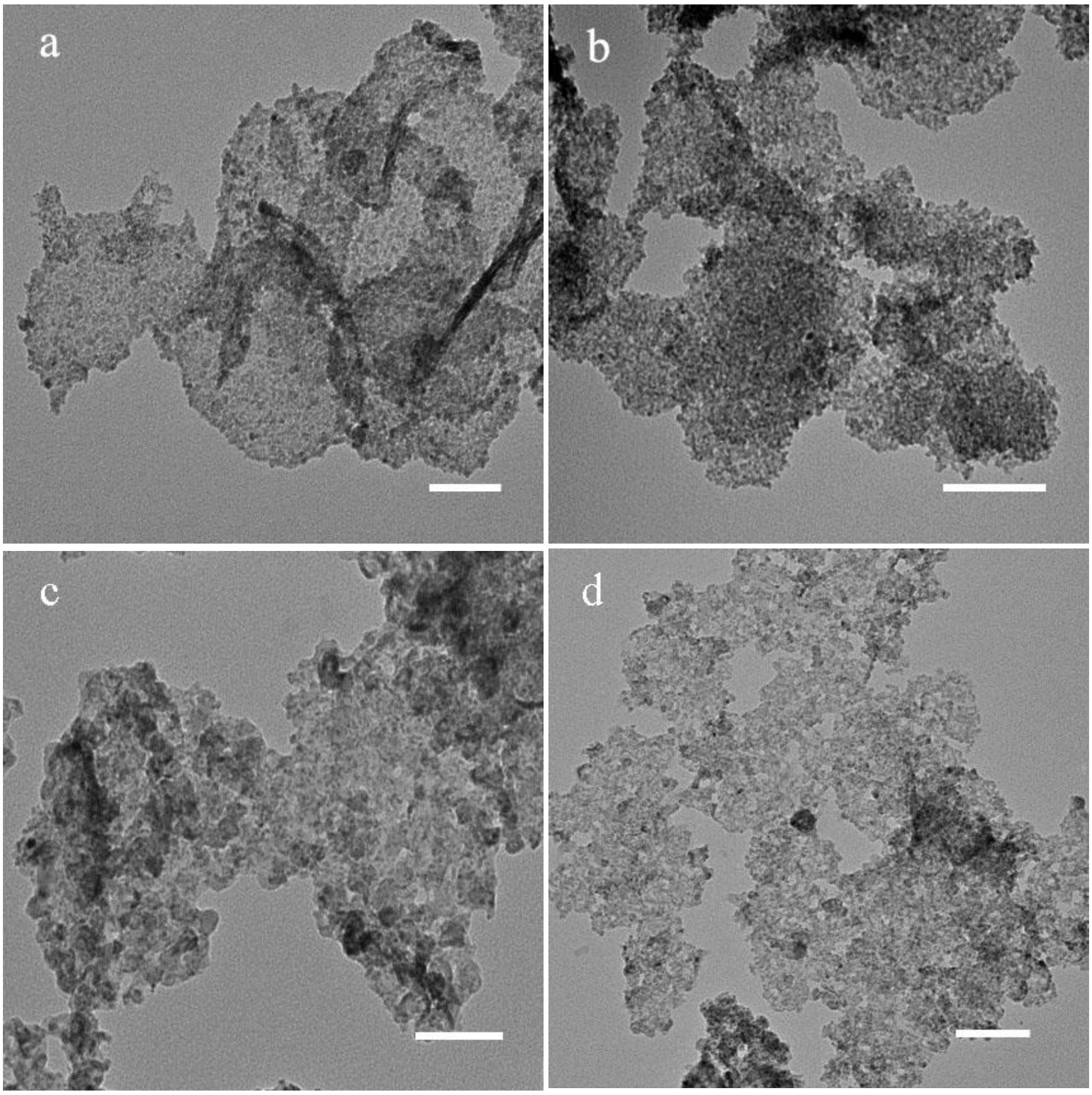


**Supplementary Figure 2.** TEM images of (a) 0H-PHNCMs; (b) 0.05H-PHNCMs; (c) 1H-PHNCMs and (d) 2.5H-PHNCMs for a large view. Scale bars: (a)-(d), 100 nm.

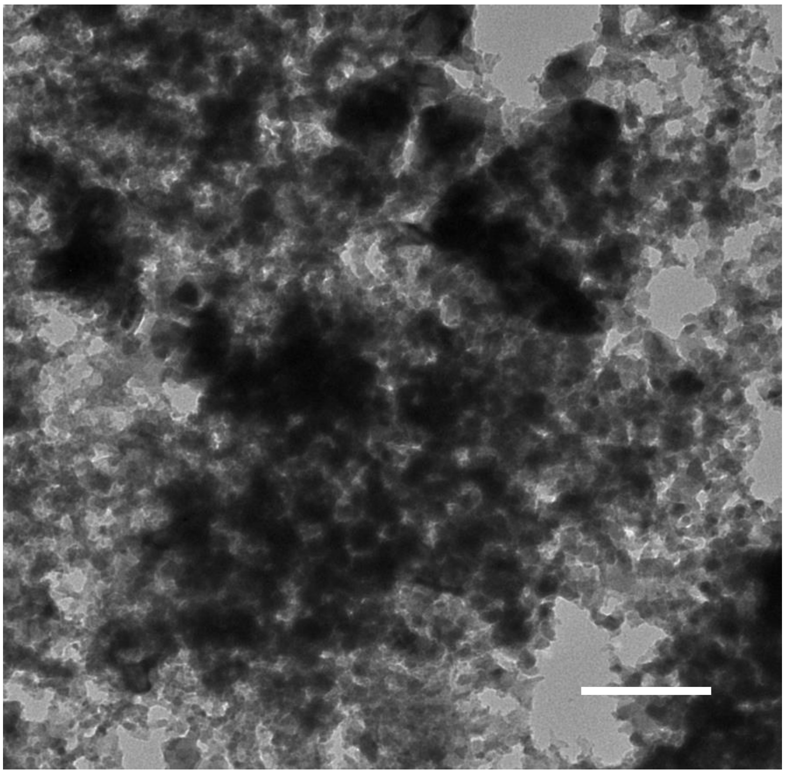

**Supplementary Figure 3.** TEM image of the corresponding products synthesized by nickel acetate and cobalt acetate directly in the same reaction system as 0H-PHNCMs, which shows some aggregations. Scale bars: 100 nm.

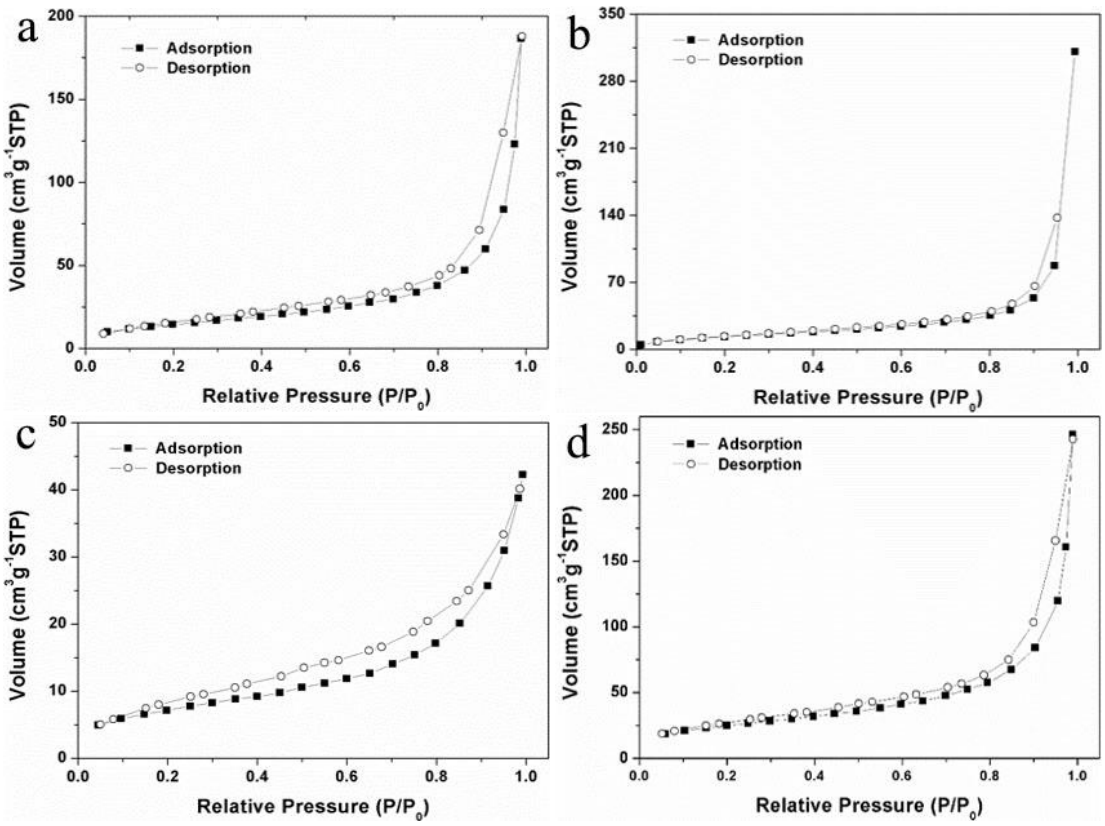


**Supplementary Figure 4.** $N_2$ adsorption–desorption isotherms measured at 77 K of (a) 0H-PHNCMs; (b) 0.05H-PHNCMs; (c) 1H-PHNCMs and (d) 2.5H-PHNCMs. The specific surface areas are calculated to be 53.35 $m^2 \cdot g^{-1}$, 26.31 $m^2 \cdot g^{-1}$, 51.95 $m^2 \cdot g^{-1}$ and 90.68 $m^2 \cdot g^{-1}$, respectively.

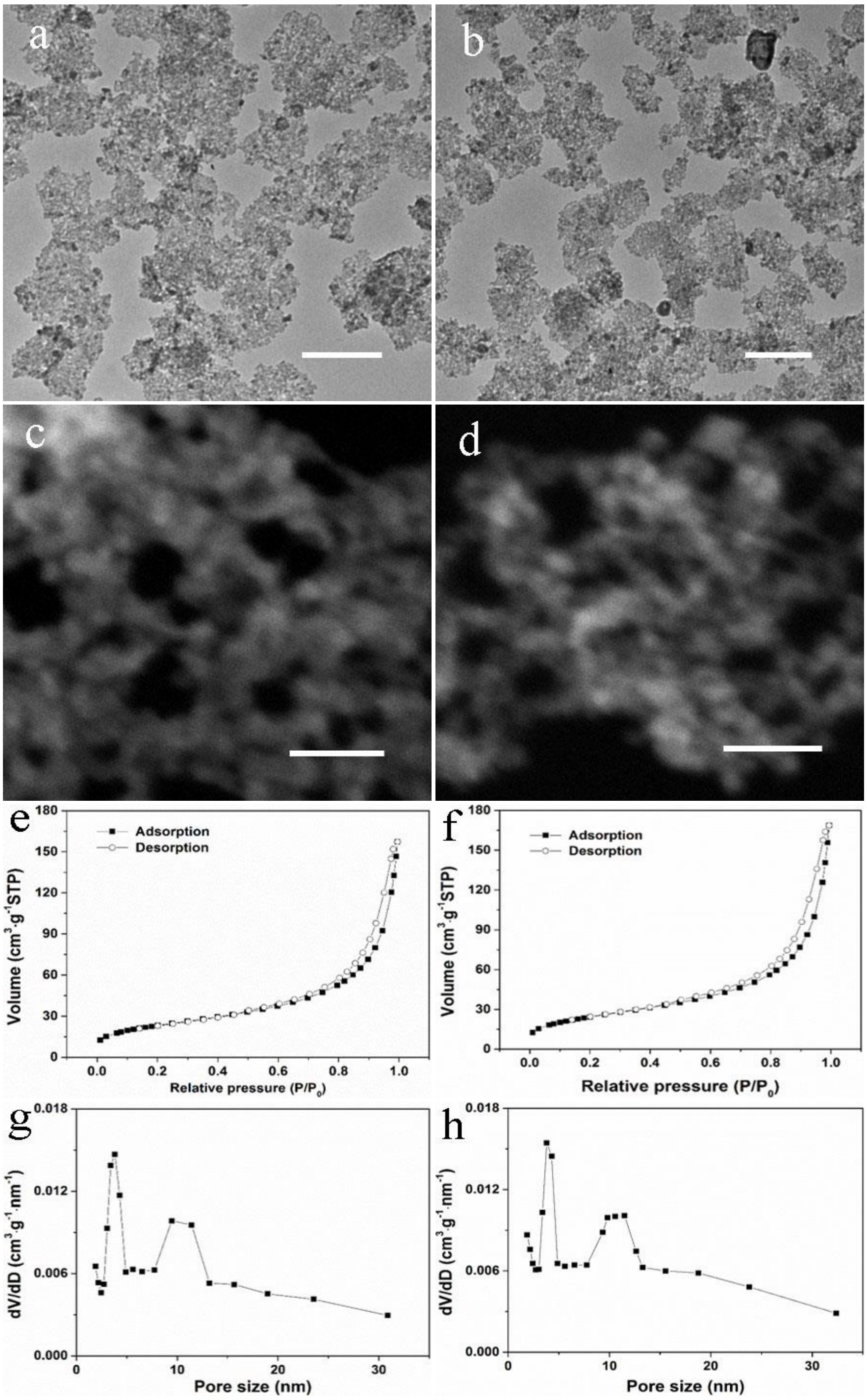


**Supplementary Figure 5.** (a) and (b) TEM images, (c) and (d) HADDF-STEM images, (e) and (f) $N_2$ adsorption–desorption isotherms measured at 77 K, (g) and (h) the corresponding pore size distribution curves of 3H- and 5H-PHNCMs. The specific surface areas were calculated to be 84.98 $m^2 \cdot g^{-1}$ and 89.36 $m^2 \cdot g^{-1}$, respectively. Scale bars in (a) and (b), 200 nm; (c) and (d), 20 nm.

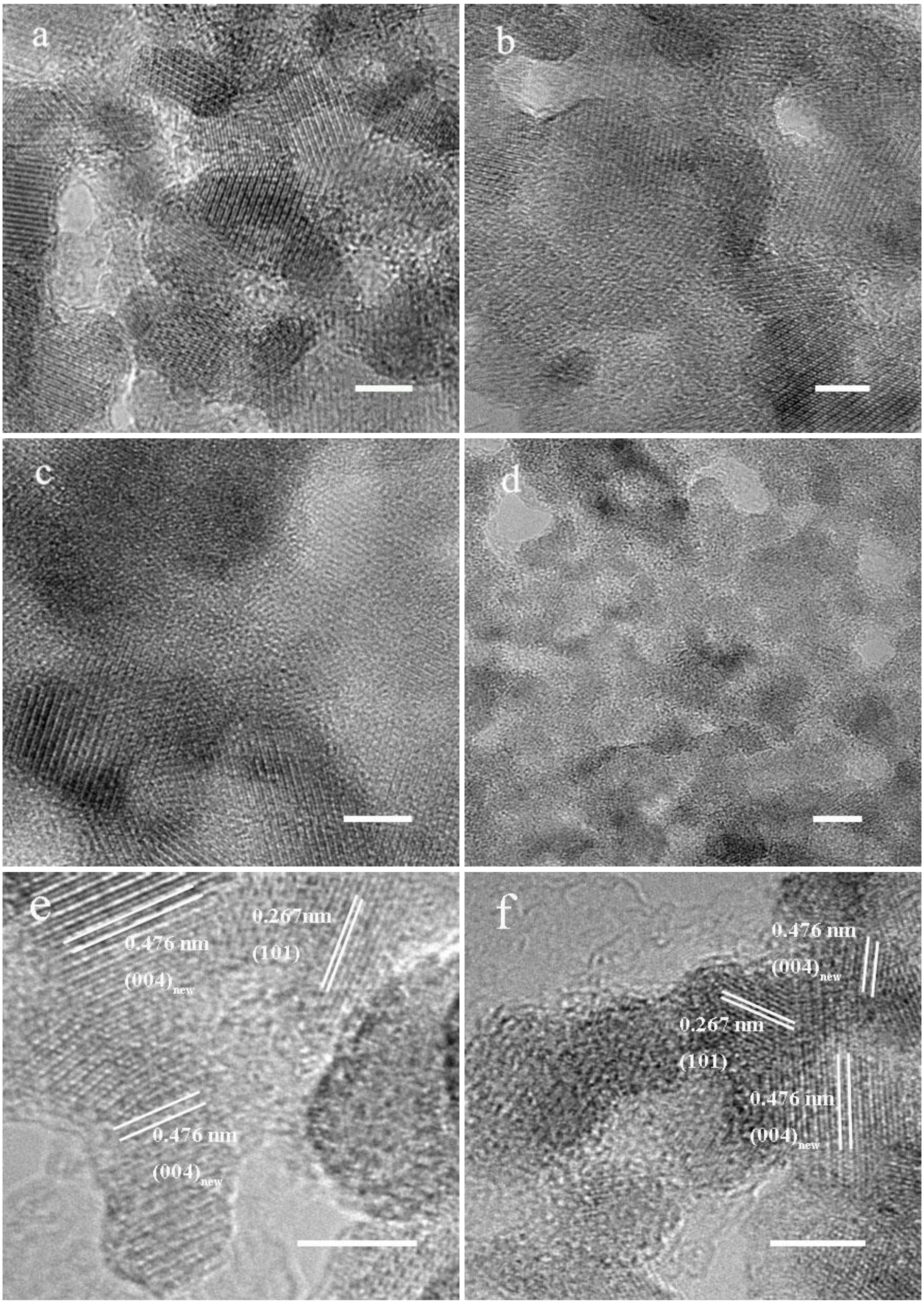


**Supplementary Figure 6.** HRTEM images of (a) 0H-, (b) 0.05H-, (c) 1H-, (d) 2.5H-, (e) 3H- and (f) 5H-PHNCMs. The five PHNCMs with HZH are composed of highly crystalline and amorphous nanostructures. Scale bars: (a)-(d), 10 nm; (e) and (f), 5 nm.

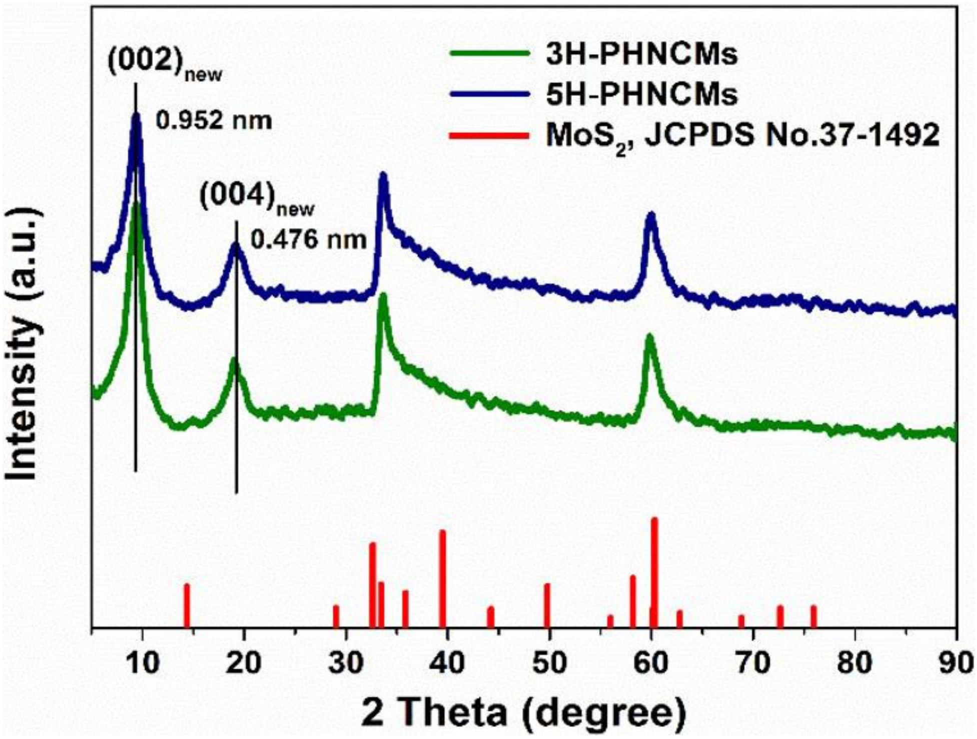


**Supplementary Figure 7.** XRD patterns of 3H- and 5H-PHNCMs. The red lines present the standard $MoS_2$ (JCPDS No. 37-1492) peaks.

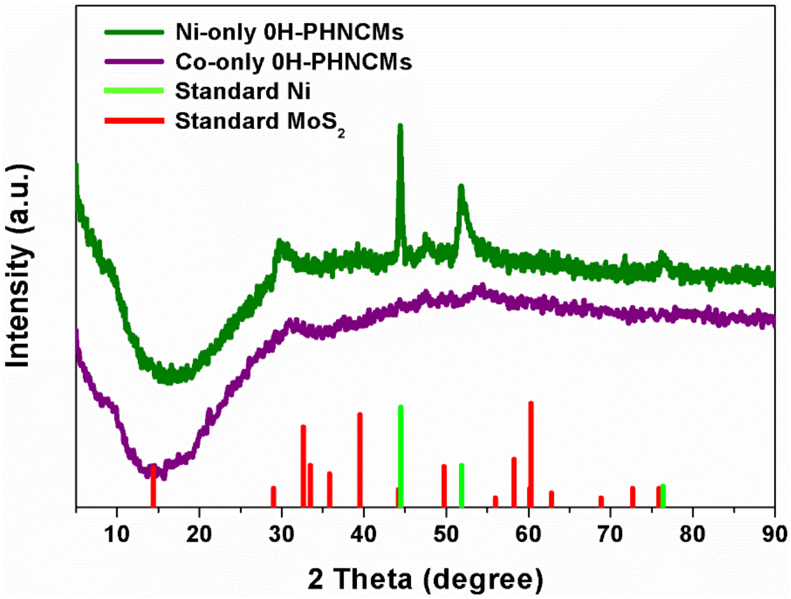


**Supplementary Figure 8.** XRD patterns of Ni-only and Co-only hybrid nanostructures synthesized in the same way as 0H-PHNCMs. The red vertical lines present the peaks of standard $MoS_2$ (JCPDS No.37-1492) and the green lines reveal the peaks of standard Ni metal (JCPDS No.04-0850).

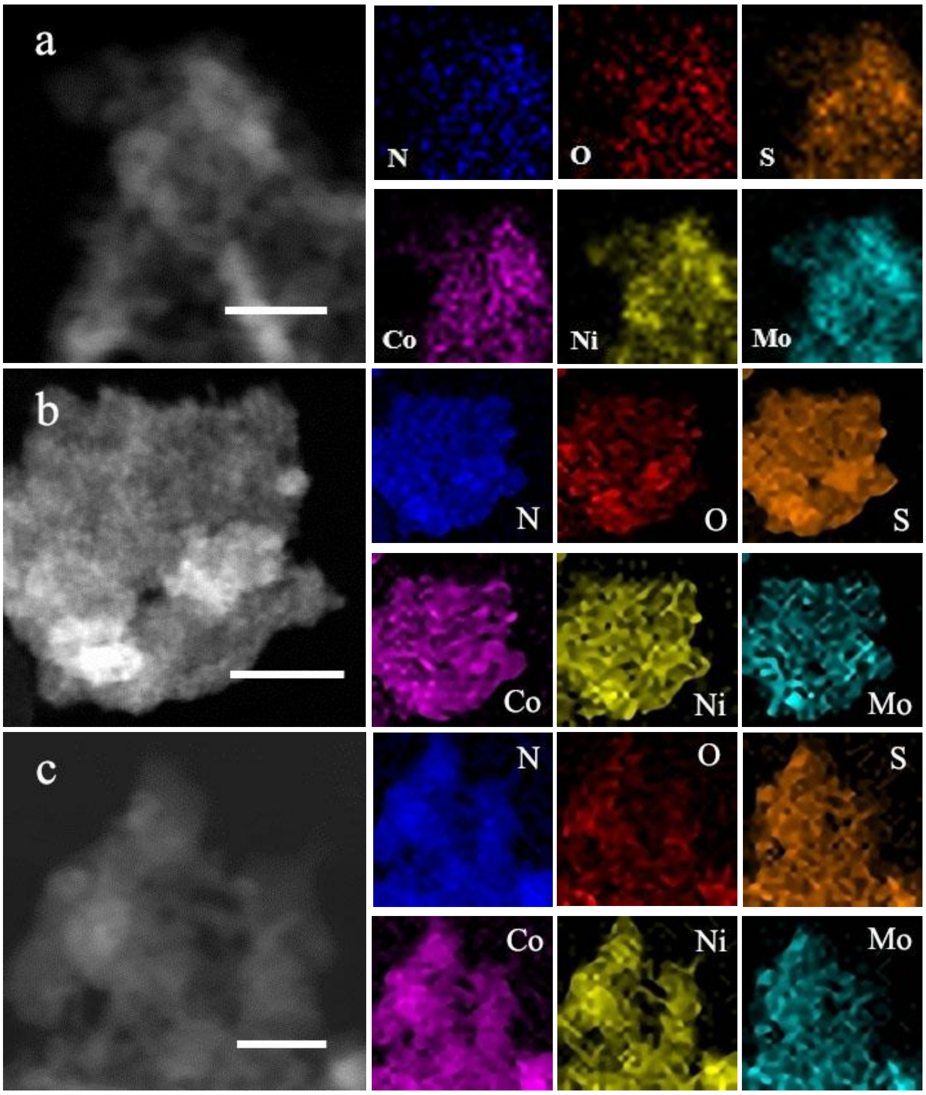


**Supplementary Figure 9.** STEM and EDX mapping spectra of (a) 0H-PHNCMs; (b) 0.05H-PHNCMs and (c) 1H-PHNCMs. Scale bars in (a)-(c): 50 nm.

**Supplementary Table 1.** Structural parameters for Co and Ni atoms in PHNCMs fitted from EXAFS data. N is the coordination number, R is the bond length, $\sigma^2$ is the Debye-Waller factor. Error bounds (accuracies) were estimated as N, ±5%; R, ±1%; $\sigma^2$, ±1%.

| **Co parameters** | | | | | **Ni parameters** | | | | |
|---|---|---|---|---|---|---|---|---|---|
| | **Path** | **N** | **R(Å)** | **$\sigma^2$ ($10^{-3}$ Å$^2$)** | | **Path** | **N** | **R(Å)** | **$\sigma^2$ ($10^{-3}$ Å$^2$)** |
| **0H-PHNCMs** | **Co-O/N** | **2.8** | **2.03** | **7.2** | **0H-PHNCMs** | **Ni-O/N** | **2.3** | **2.05** | **6.1** |
| | **Co-Co** | **4.0** | **2.44** | **9.3** | | **Ni-Ni** | **5.6** | **2.48** | **6.5** |
| **0.05H-PHNCMs** | **Co-O/N** | **3.4** | **2.07** | **5.9** | **0.05H-PHNCMs** | **Ni-O/N** | **3.5** | **2.05** | **5.2** |
| | **Co-Co** | **2.3** | **2.43** | **9.0** | | **Ni-Ni** | **1.6** | **2.41** | **9.9** |
| **1H-PHNCMs** | **Co-O/N** | **2.5** | **2.02** | **7.0** | **1H-PHNCMs** | **Ni-O/N** | **2.6** | **2.05** | **4.4** |
| | **Co-Co** | **3.4** | **2.43** | **9.2** | | **Ni-Ni** | **1.8** | **2.41** | **9.4** |
| **2.5H-PHNCMs** | **Co-O/N** | **2.3** | **2.01** | **6.8** | **2.5H-PHNCMs** | **Ni-O/N** | **2.8** | **2.07** | **4.6** |
| | **Co-Co** | **3.3** | **2.42** | **9.1** | | **Ni-Ni** | **1.4** | **2.41** | **9.5** |
| **Co foil** | **Co-Co** | **12** | **2.49** | **6.8** | **Ni foil** | **Ni-Ni** | **12** | **2.48** | **6.2** |
| **CoO** | **Co-O** | **6** | **2.10** | **4.3** | **NiO** | **Ni-O** | **6** | **2.08** | **4.3** |
| | **Co-Co** | **12** | **2.42** | **7.6** | | **Ni-Ni** | **12** | **2.95** | **5.6** |

**Supplementary Table 2.** Structural parameters for Mo atoms in PHNCMs fitted from EXAFS data. Error bounds (accuracies) were estimated as N, ±5%; R, ±1%; $\sigma^2$, ±1%.

| Samples | Path | N | Bond length | $\sigma^2(10^{-3}$ Å$^2)$ |
|---|---|---|---|---|
| 0H-PHNCMs | Mo-O/N | 1.7 | 1.74 | 1.4 |
| | Mo-S | 2.4 | 2.30 | 6.7 |
| | Mo-Mo | 0.6 | 2.79 | 5.0 |
| | Mo-Mo | 0.5 | 3.18 | 7.0 |
| 0.05H-PHNCMs | Mo-O/N | 1.6 | 1.76 | 1.0 |
| | Mo-S | 2.6 | 2.31 | 6.3 |
| | Mo-Mo | 0.8 | 2.77 | 4.8 |
| | Mo-Mo | 0.6 | 3.18 | 6.1 |
| 1H-PHNCMs | Mo-O/N | 0.9 | 1.77 | 1.3 |
| | Mo-S | 3.4 | 2.33 | 4.9 |
| | Mo-Mo | 1.8 | 2.79 | 6.3 |
| 2.5H-PHNCMs | Mo-O/N | 1.3 | 1.74 | 1.5 |
| | Mo-S | 2.9 | 2.33 | 5.9 |
| | Mo-Mo | 1.0 | 2.78 | 5.7 |
| 2H-$MoS_2$ foil | Mo-S | 6 | 2.41 | 3.2 |
| | Mo-Mo | 6 | 3.16 | 3.6 |

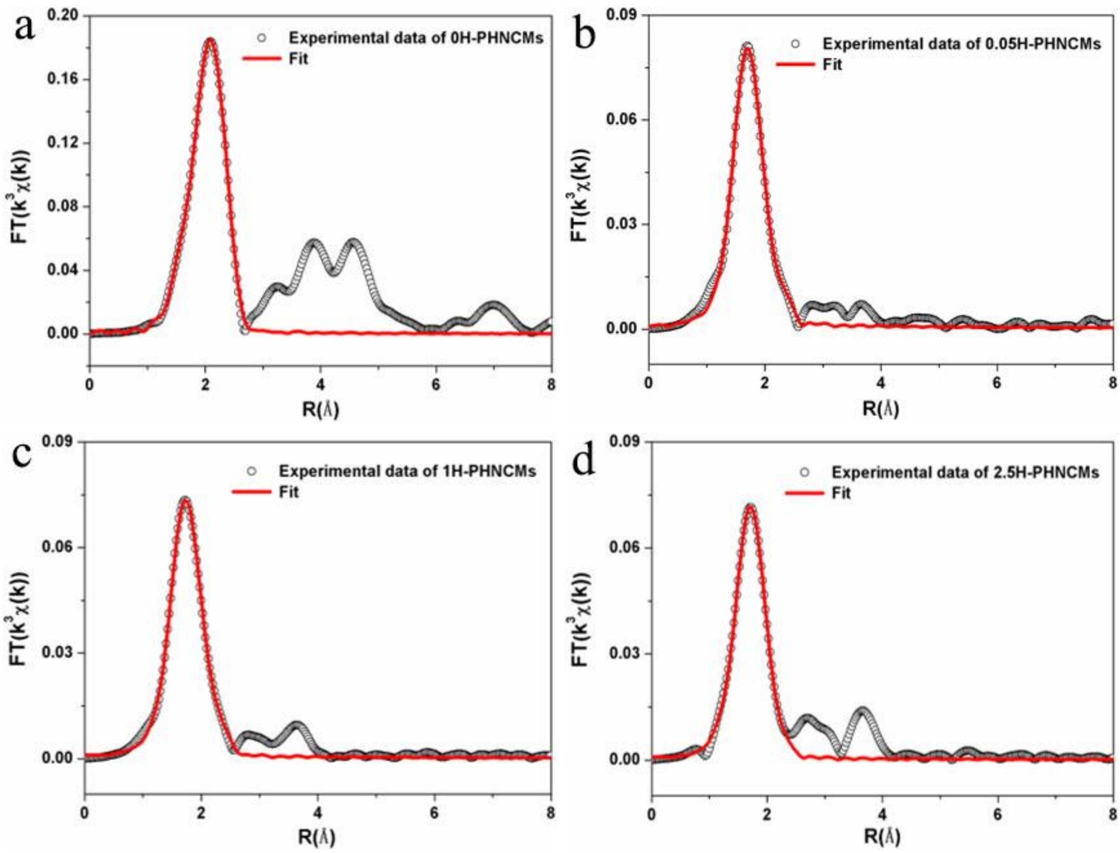


**Supplementary Figure 10.** Comparison between experimental data and the fitting curves of PHNCMs for Ni K-edge.

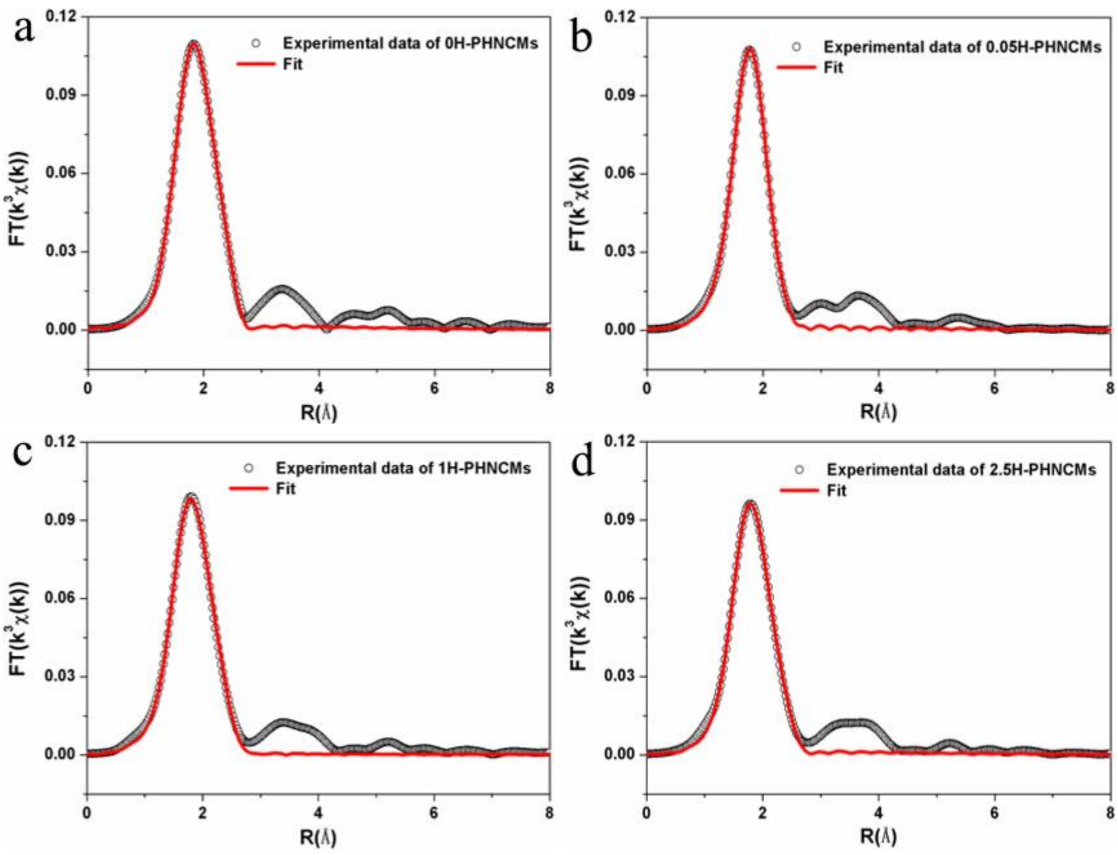


**Supplementary Figure 11.** Comparison between experimental data and the fitting curves of PHNCMs for Co K-edge.

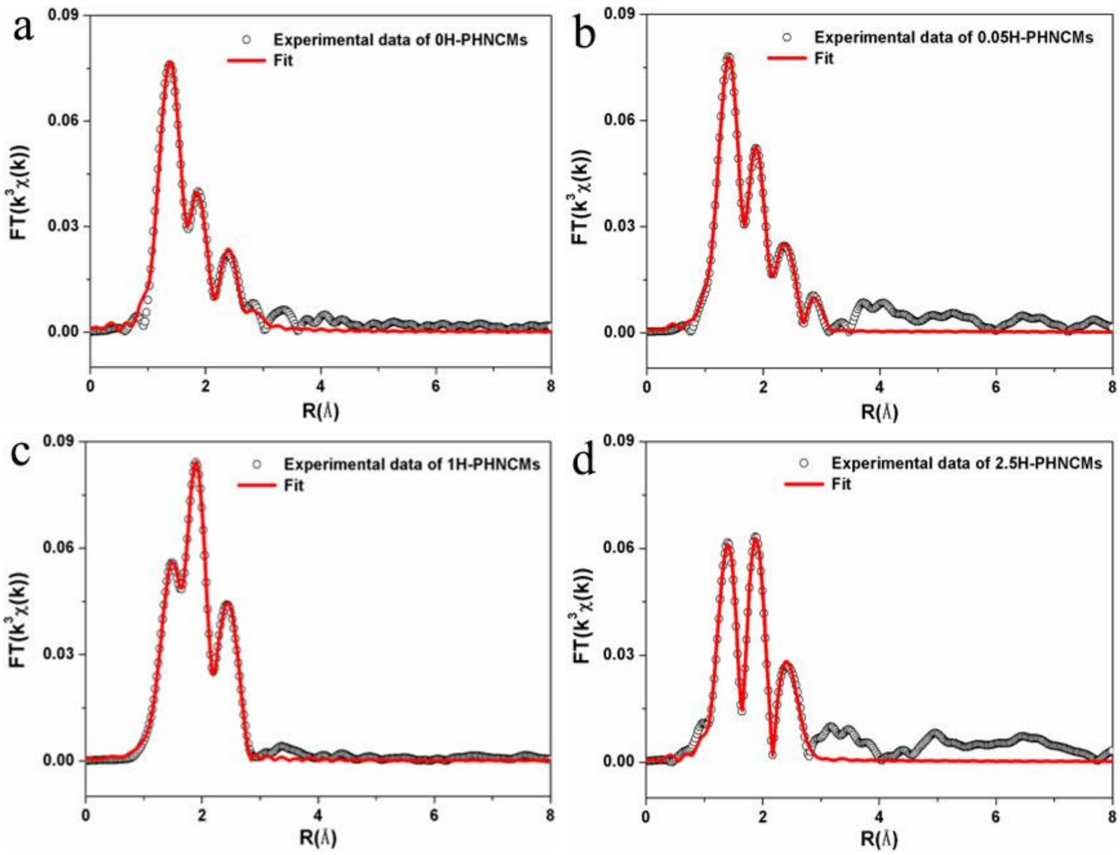


**Supplementary Figure 12.** Comparison between experimental data and the fitting curves of PHNCMs for Mo K-edge.

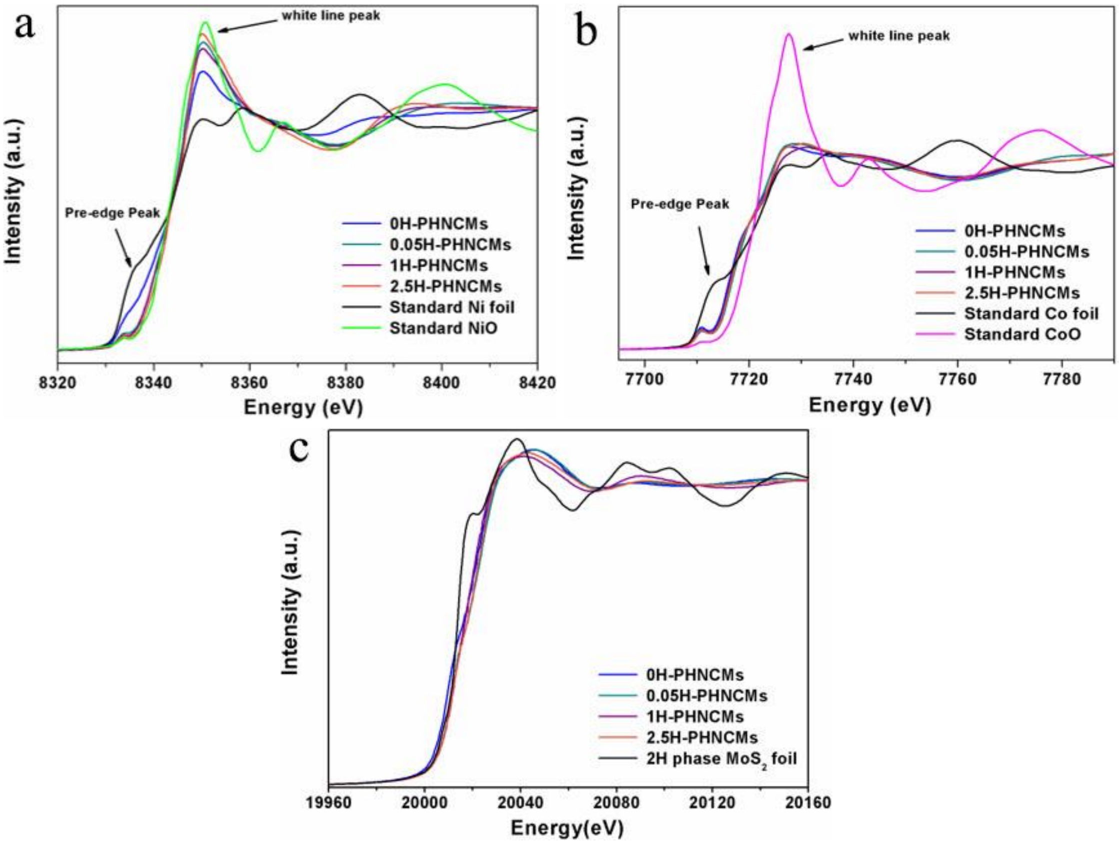


**Supplementary Figure 13.** The normalized XANES spectra from EXAFS at the (a) Ni, (b) Co and (c) Mo K-edge of the PHNCMs and Co foil, CoO, Ni foil, NiO, 2H phase $MoS_2$ foil as contrastive samples.

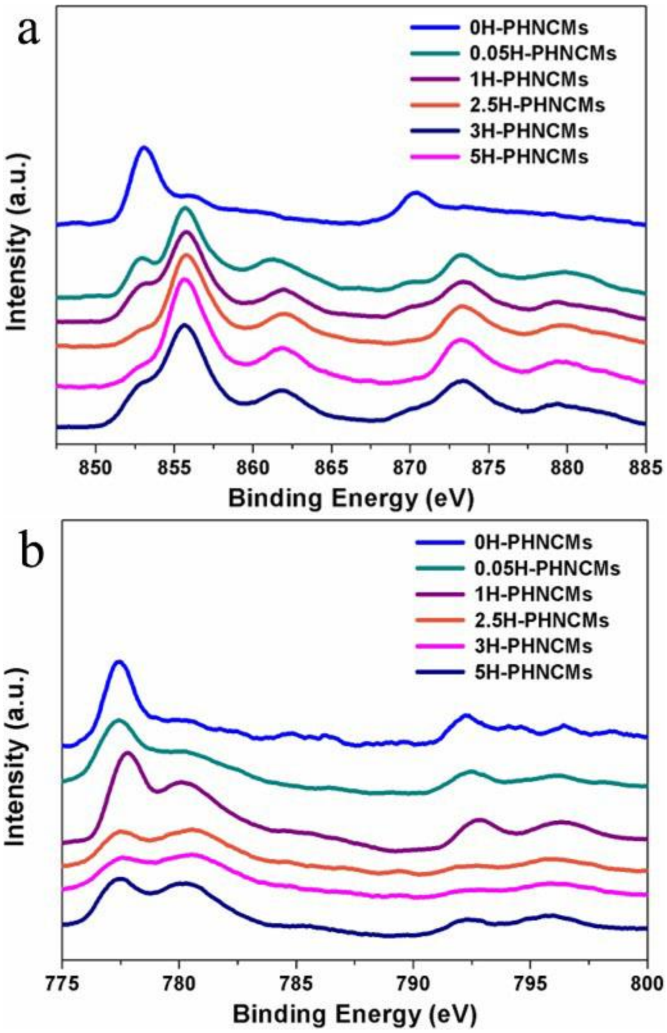


**Supplementary Figure 14.** The smoothing XPS spectra showing the binding energies of (a) Ni and (b) Co in PHNCMs.

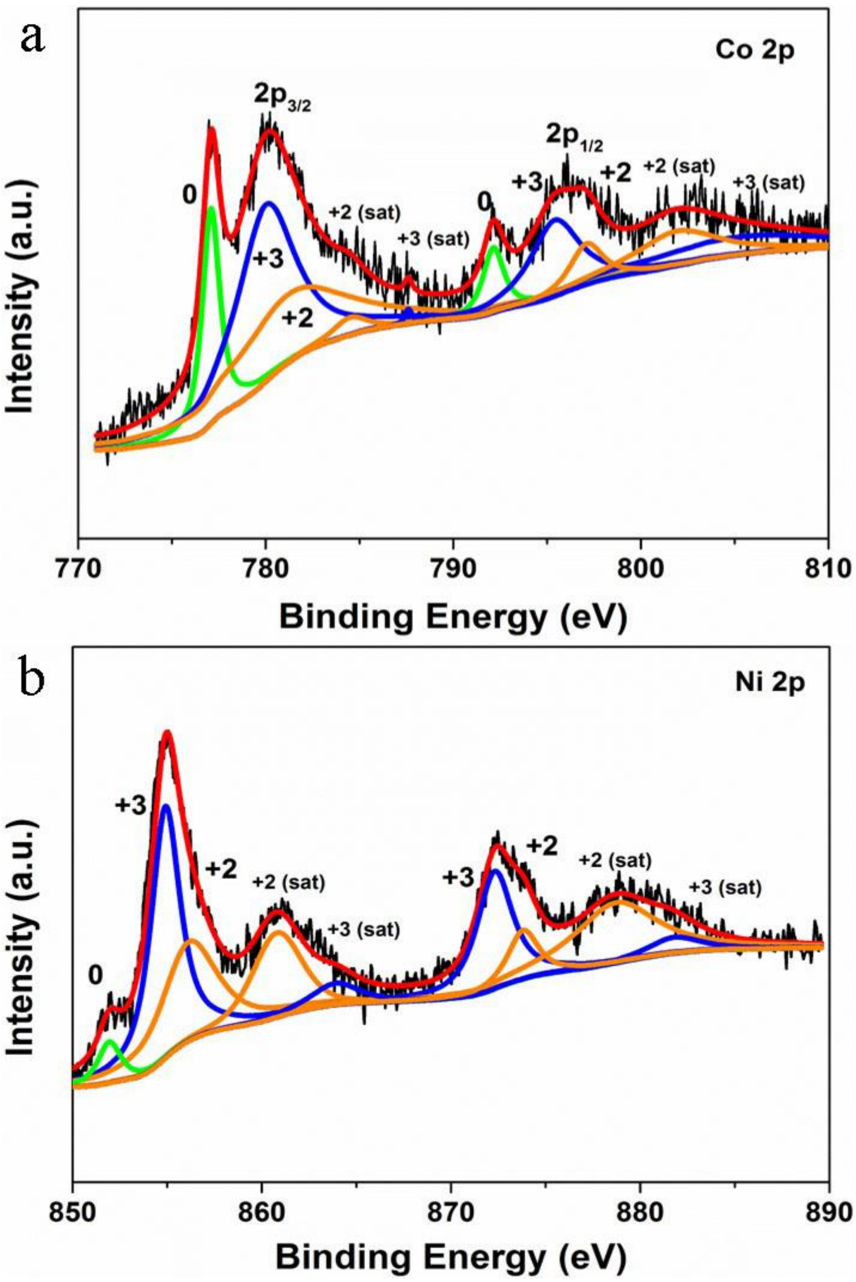


**Supplementary Figure 15.** The fitting results of XPS spectra of (a) Co 2p and (b) Ni 2p orbitals in 2.5H-PHNCMs.

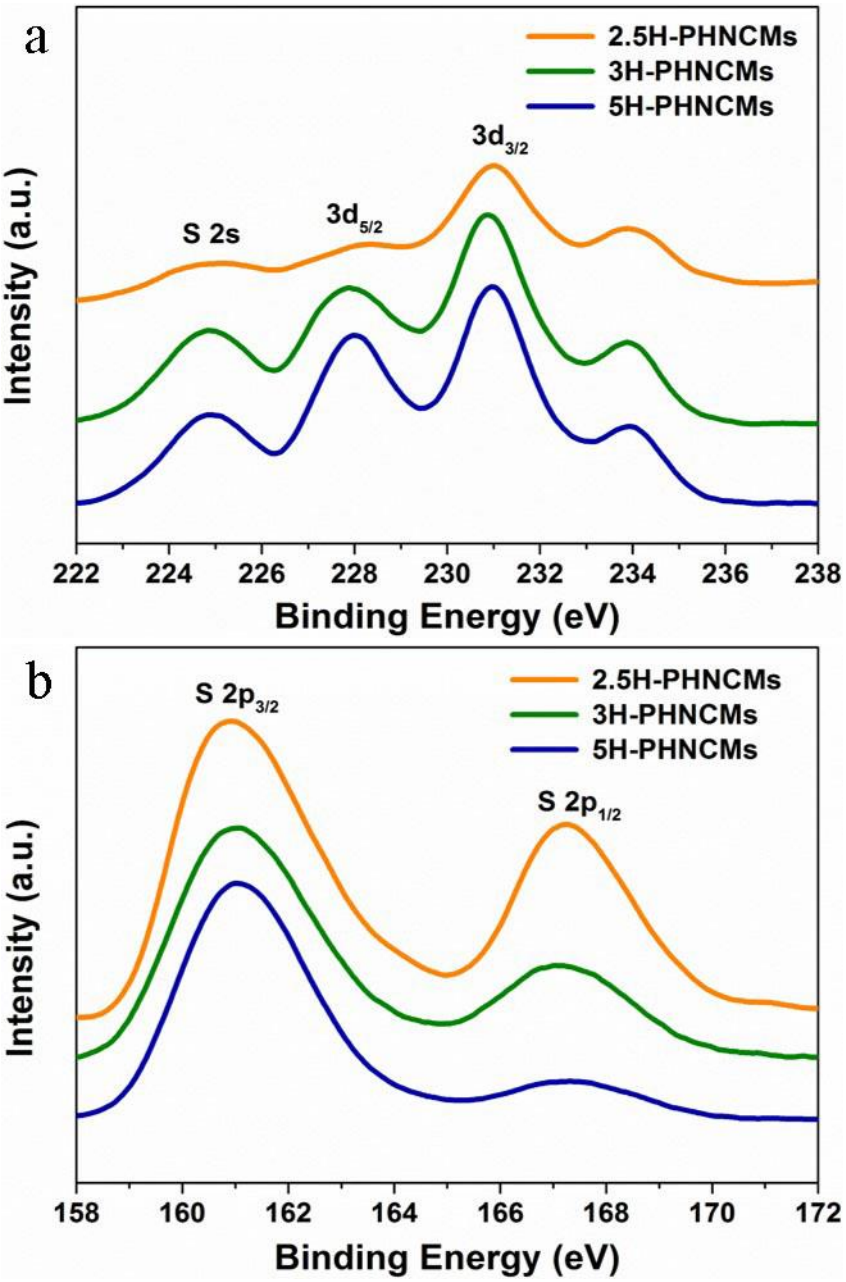


**Supplementary Figure 16.** The smoothing XPS spectra of (a) Mo and (b) S in 2.5H-, 3H- and 5H-PHNCMs.

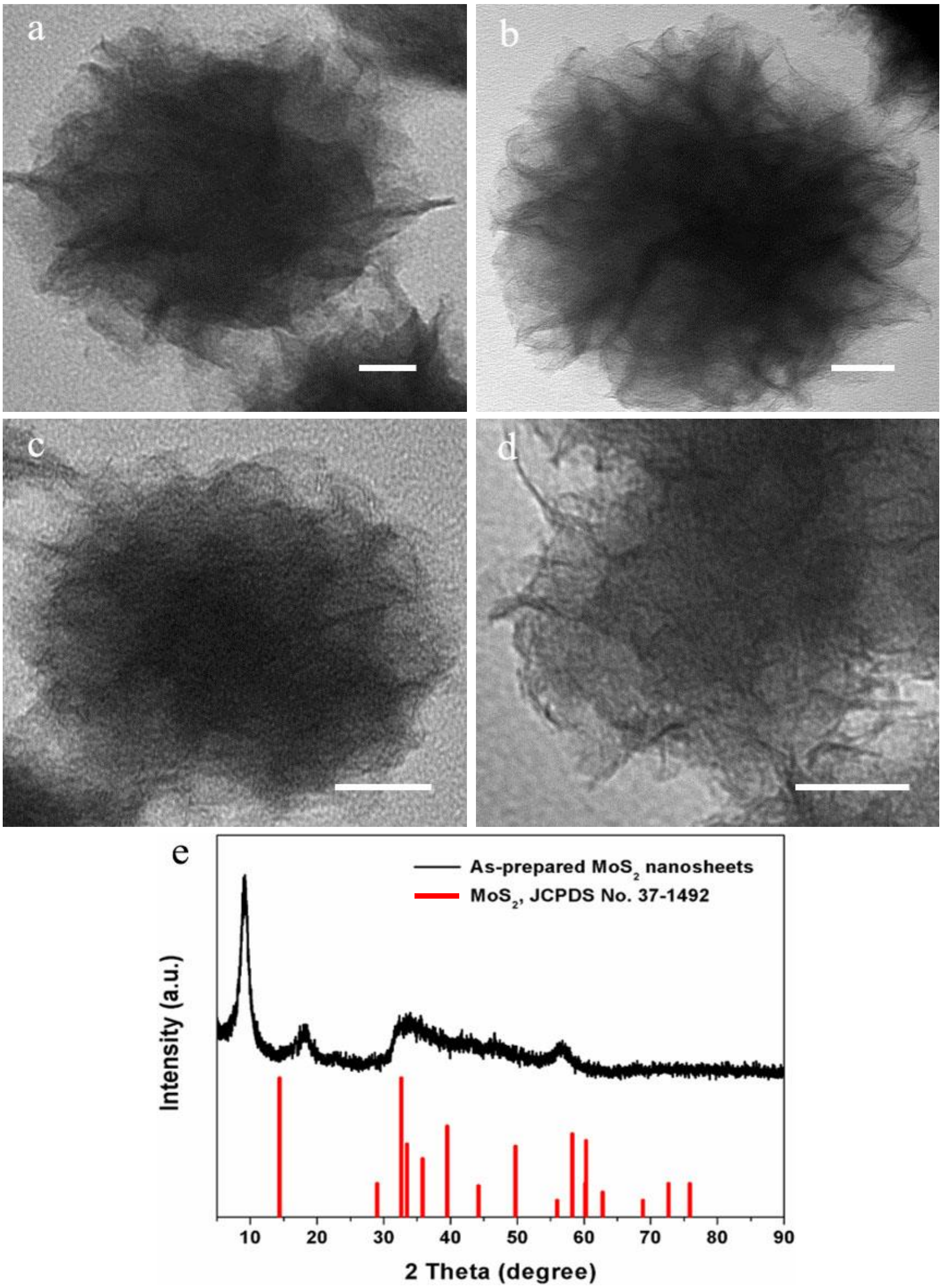


**Supplementary Figure 17.** (a)-(d) TEM images of 0H-$MoS_2$, 0.05H-$MoS_2$, 1H-$MoS_2$ and 2.5H-$MoS_2$. (e) XRD pattern of as-prepared $MoS_2$ nanosheets with different amount of HZH. The two peaks below 20° demonstrate the enlarged interlayer spacing emerged as compared to pristine $MoS_2$, which is in accordance with that of PHNCMs with HZH. Scale bars: (a)-(d), 20 nm.

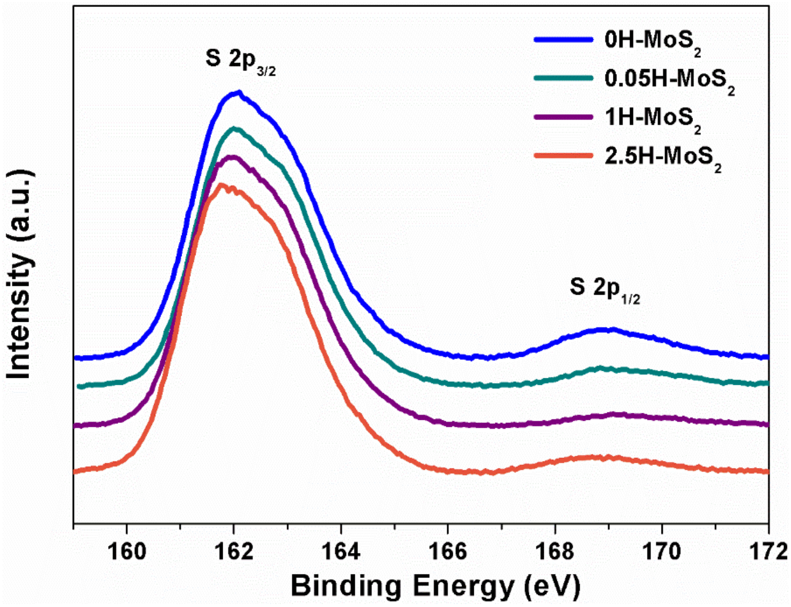


**Supplementary Figure 18.** XPS spectrum of S 2p orbitals in $MoS_2$ synthesized in the same way as PHNCMs. The difference of binding energies between peaks of 0H-$MoS_2$ and 2.5H-$MoS_2$ is around 0.2 eV.

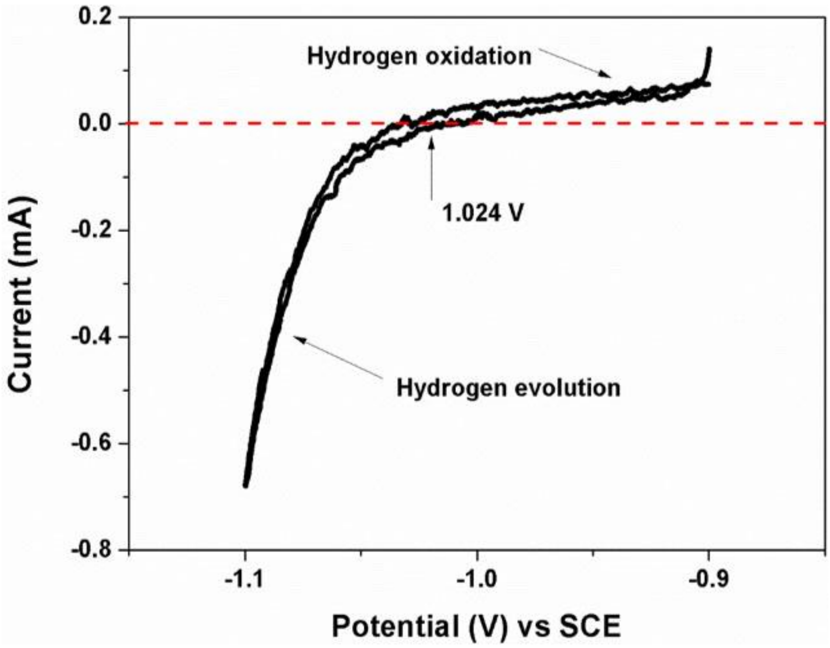


**Supplementary Figure 19.** RHE calibration plot. In 1 M KOH, E (RHE) = E (SCE) + 1.024 V.

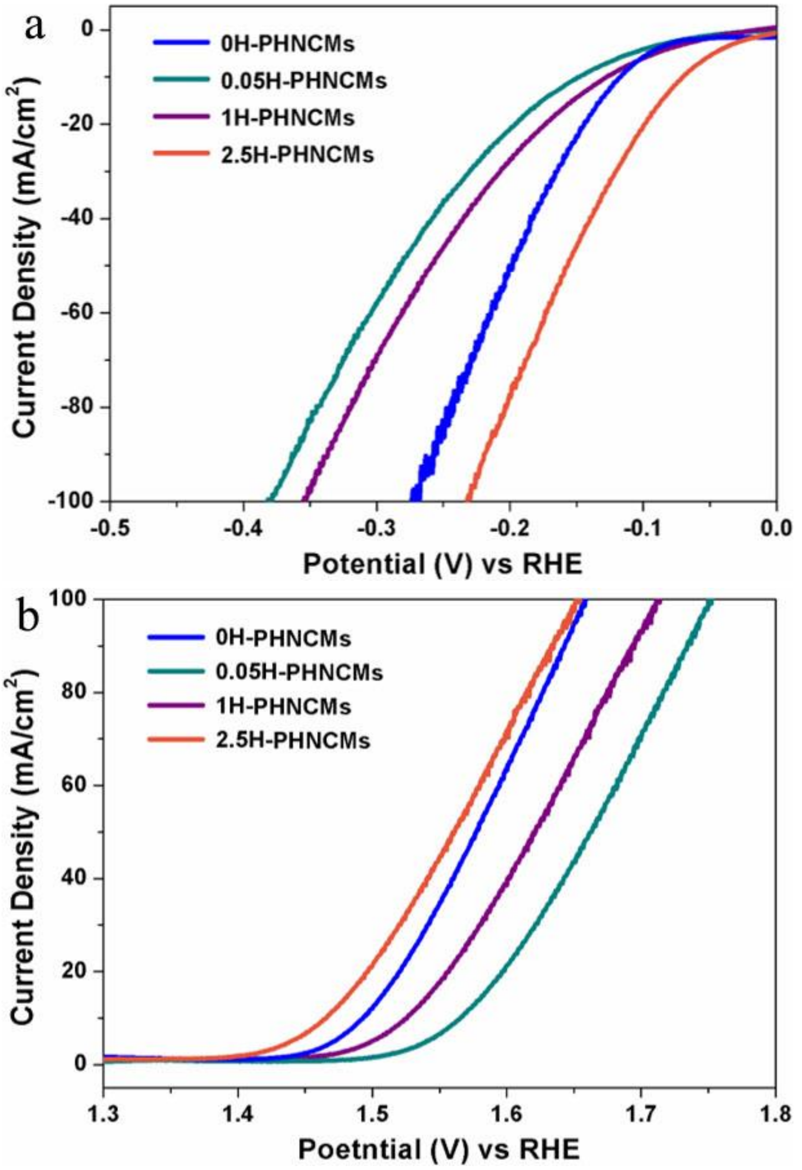


**Supplementary Figure 20.** The comparison of polarization curves for (a) HER and (b) OER of PHNCMs measured at a scan rate of 5 $mV \cdot s^{-1}$ in 1 M KOH solution.

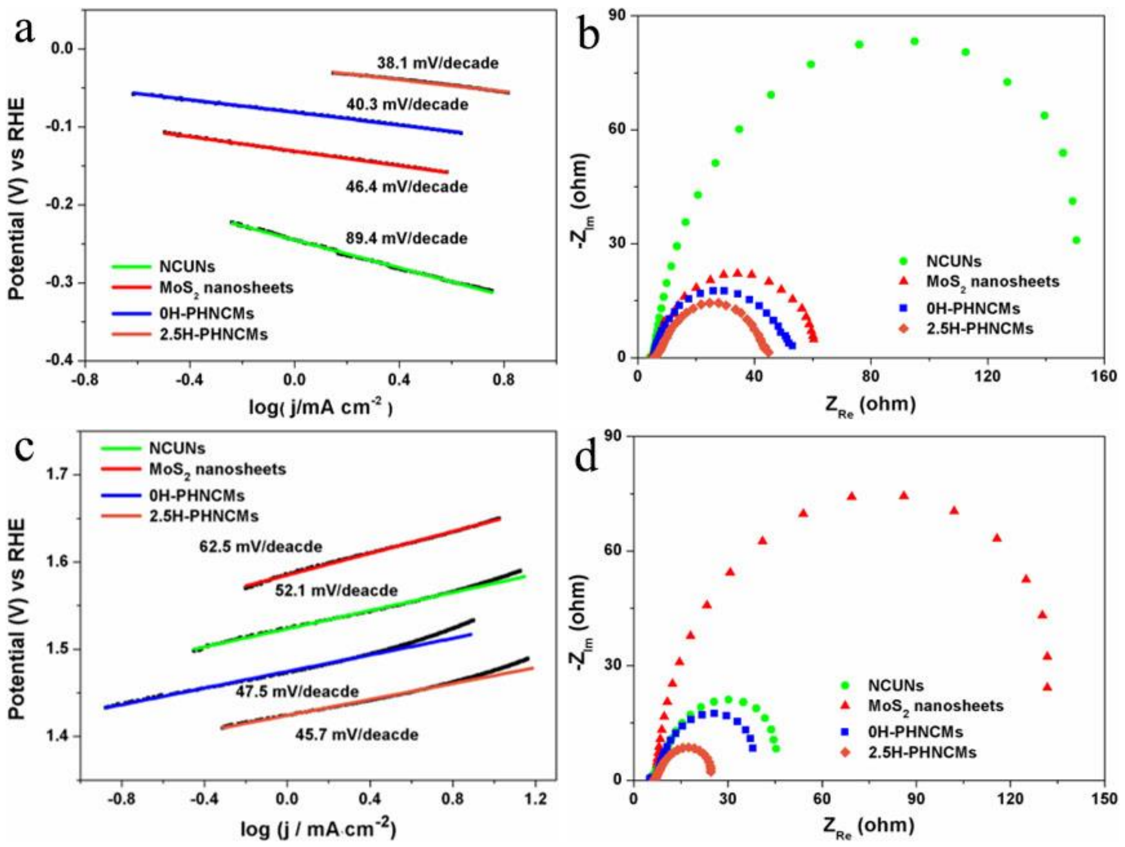


**Supplementary Figure 21.** (a) and (c) Tafel plots measured at a scan rate of 1 mV·s$^{-1}$; (b) and (d) EIS Nyquist plots of 2.5H-PHNCMs and 0H-PHNCMs, NCUNs and $MoS_2$ nanosheets at a current density of 1 mA·cm$^{-2}$ for HER and OER.

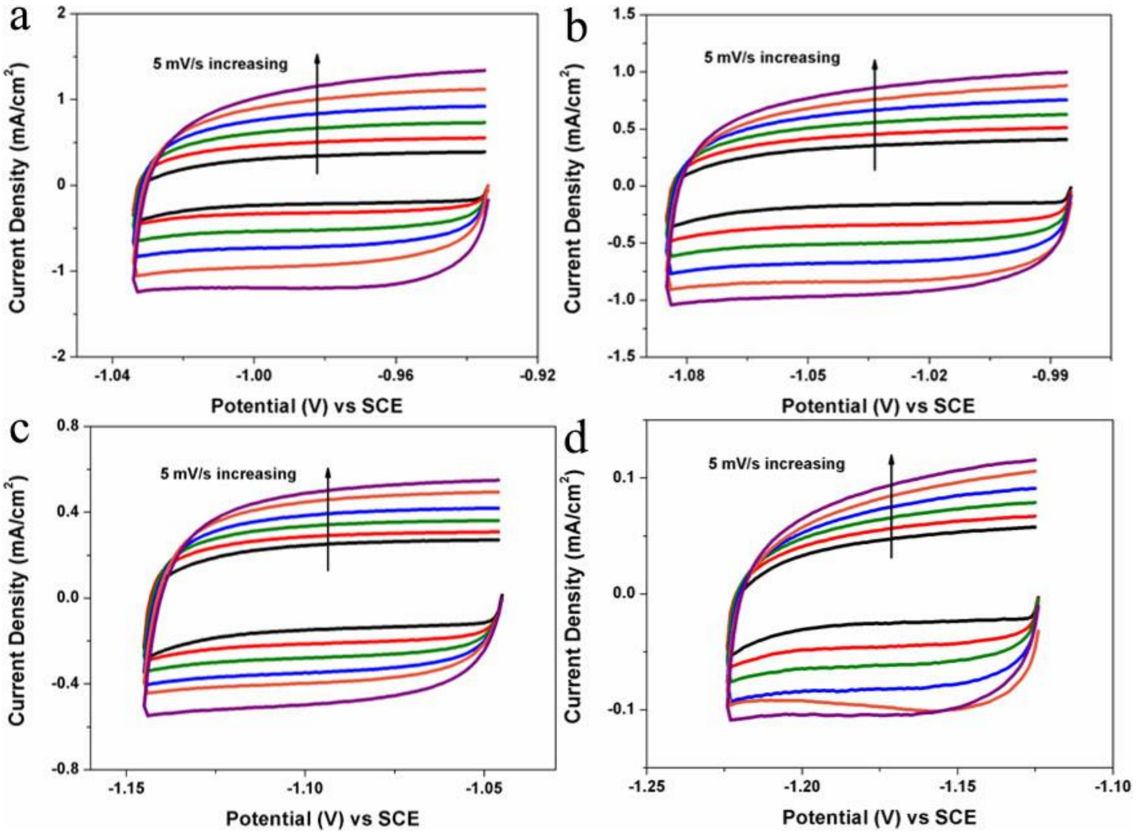


**Supplementary Figure 22.** HER cyclic voltammetry at the scan rates of 5, 10, 15, 20, 25, 30 mV·s$^{-1}$ for ECSA of (a) 2.5H-PHNCMs, (b) 0H-PHNCMs, (c) $MoS_2$ nanosheets and (d) NUCNs. The current densities at the average potential in the selected range were plotted as a function of the scan rates and the slope of the linear fit could be calculated as the $C_{dl}$.

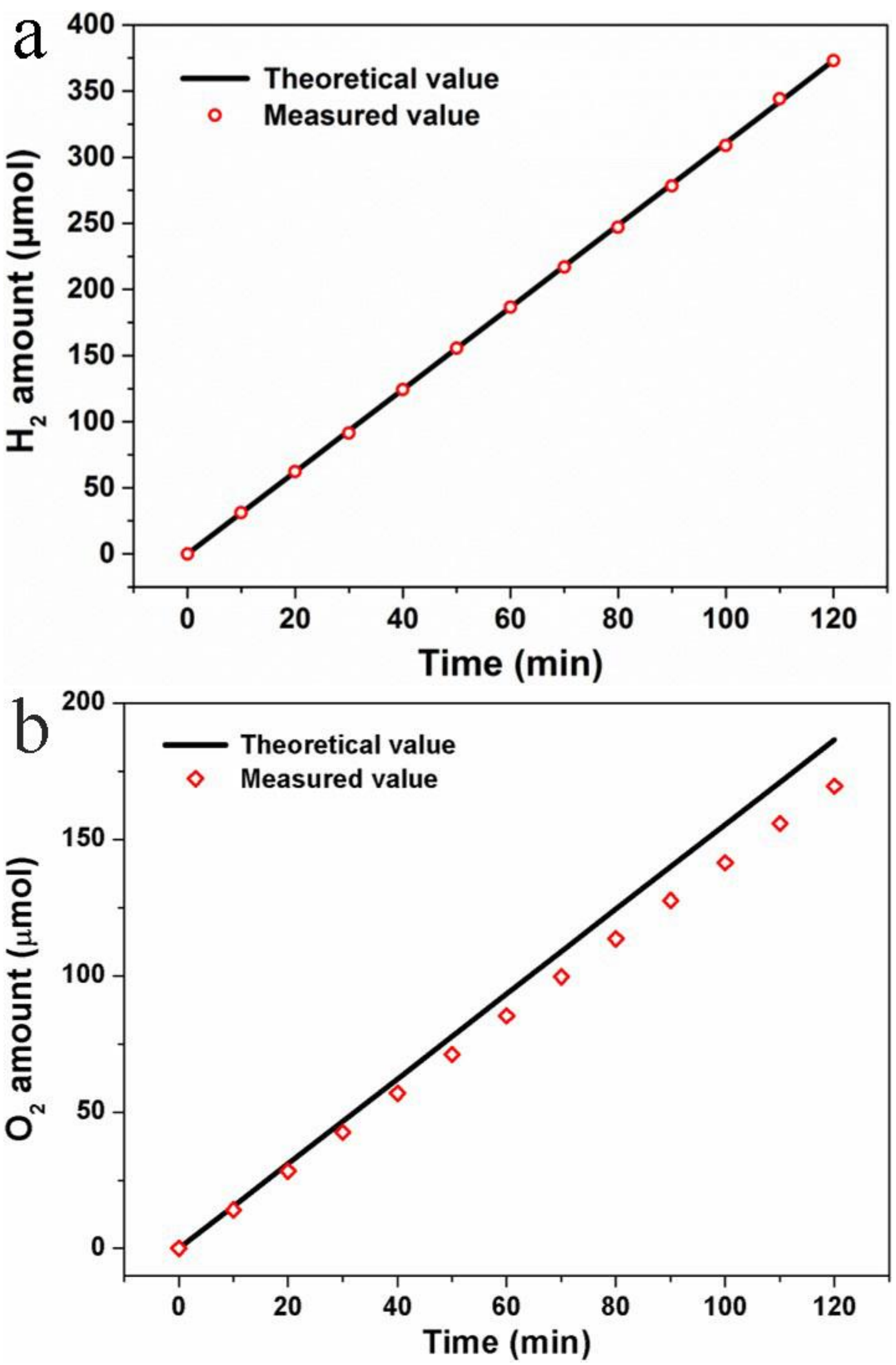


**Supplementary Figure 23.** (a) and (b) Electrocatalytic Faradaic efficiencies of HER and OER using 2.5H-PHNCMs as the active material on a piece of carbon fiber paper (effective electrode area: 0.5 cm×1 cm) at a current density of 20 mA·cm$^{-2}$ and measured for 120 min.

**Supplementary Table 3.** The percent proportions of Co, Ni and Mo in PHNCMs.

| | **0H-PHNCMs** | **0.05H-PHNCMs** | **1H-PHNCMs** | **2.5H-PHNCMs** |
|---|---|---|---|---|
| **Elements** | **Atomic%** | | | |
| **Co** | **20.92** | **19.89** | **19.87** | **19.15** |
| **Ni** | **23.93** | **21.67** | **21.05** | **20.80** |
| **Mo** | **12.64** | **12.71** | **12.76** | **12.92** |

**Supplementary Table 4.** The theoretical values, measured values and Faradaic

efficiencies of OER at different periods over 2.5H-PHNCMs. The average Faradaic efficiency was 91.23%.

| Time (min) | 10 | 20 | 30 | 40 | 50 | 60 | 70 | 80 | 90 | 100 | 110 | 120 |
|---|---|---|---|---|---|---|---|---|---|---|---|---|
| Theoretical value (μmol) | 15.55 | 31.09 | 46.64 | 62.19 | 77.73 | 93.28 | 108.83 | 124.37 | 139.92 | 155.46 | 171.01 | 186.56 |
| Measured value (μmol) | 14.12 | 28.36 | 42.58 | 56.90 | 71.12 | 85.26 | 99.58 | 113.55 | 127.61 | 141.47 | 155.79 | 169.58 |
| Faradiac effiency (%) | 90.8 | 91.2 | 91.3 | 91.5 | 91.5 | 91.4 | 91.5 | 91.3 | 91.2 | 91 | 91.1 | 90.9 |

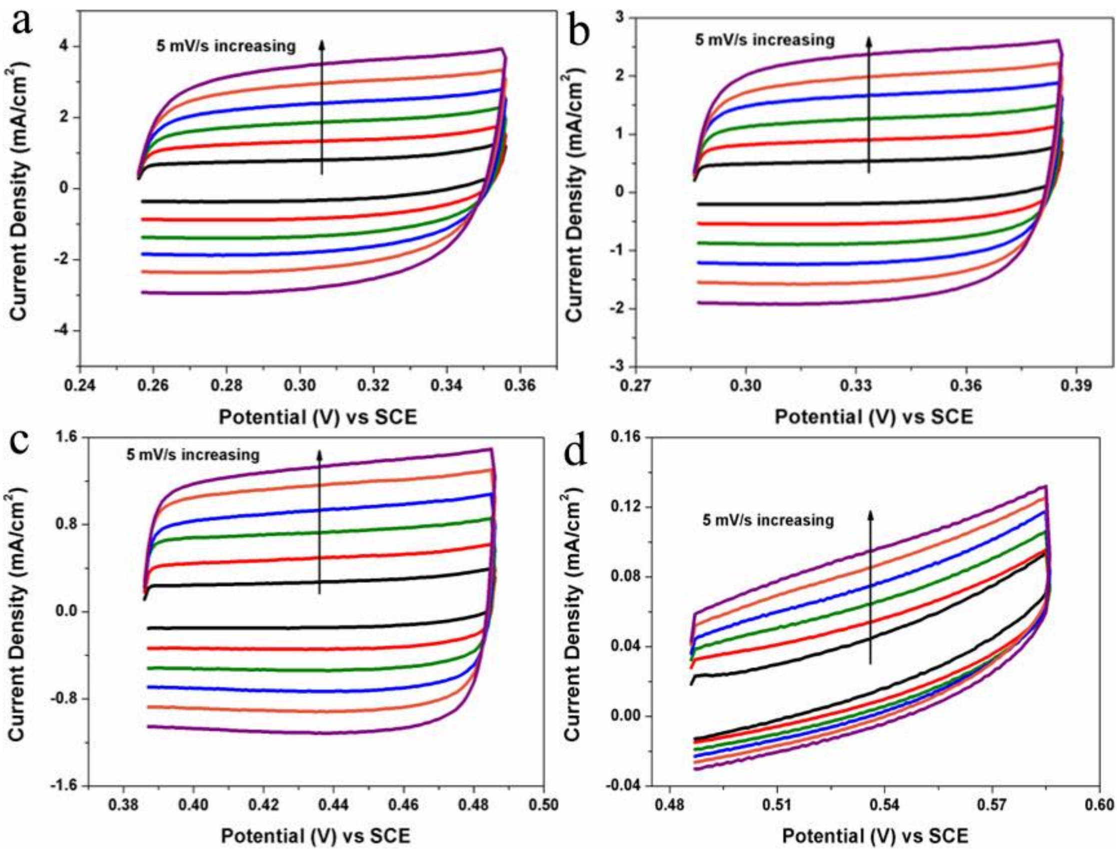


**Supplementary Figure 24.** OER cyclic voltammetry at the scan rates of 5, 10, 15, 20, 25, 30 mV·s$^{-1}$ for ECSA of (a) 2.5H-PHNCMs, (b) 0H-PHNCMs, (c) NUCNs and (d) $MoS_2$ nanosheets. The current densities at the average potential in the selected range were plotted as a function of the scan rates and the slope of the linear fit could be calculated as the $C_{dl}$.

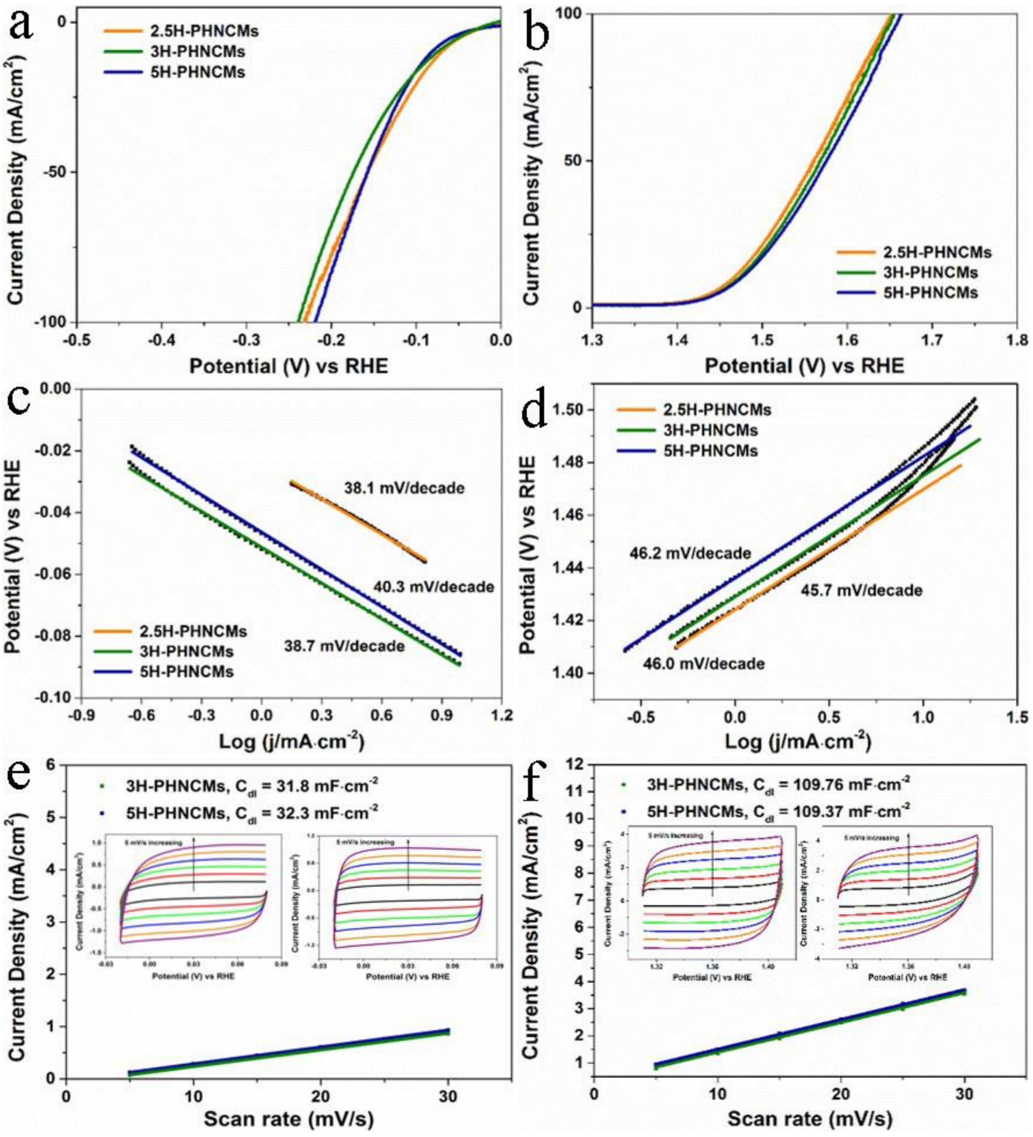


**Supplementary Figure 25.** (a) and (b) Polarization curves of 2.5H-, 3H- and 5H-PHNCMs for HER and OER measured at a scan rate of 5 mV·$s^{-1}$ in 1 M KOH; (c) and (d) the corresponding Tafel plots for HER and OER; (e) and (f) the fitting plots showing $C_{dl}$ for HER and OER. Inset: the corresponding CV scan.

**Supplementary Table 5.** Evaluation indexes of 2.5H-, 3H- and 5H-PHNCMs for HER and OER electrocatalytic activities.

| **Electrocatalytic reaction** | **Samples** | **Overpotential at 10 mA/cm$^2$ ( V vs RHE)** | **Tafel slope (mV/ decade )** | **The double-layer capacitance (mF/cm$^2$)** |
|---|---|---|---|---|
| **HER** | **2.5H-PHNCMs** | **0.07** | **38.1** | **32.3** |
| | **3H-PHNCMs** | **0.075** | **38.7** | **31.8** |
| | **5H-PHNCMs** | **0.08** | **40.3** | **32.3** |
| **OER** | **2.5H-PHNCMs** | **1.465** | **45.7** | **108.1** |
| | **3H-PHNCMs** | **1.472** | **46.0** | **109.8** |
| | **5H-PHNCMs** | **1.474** | **46.2** | **109.4** |

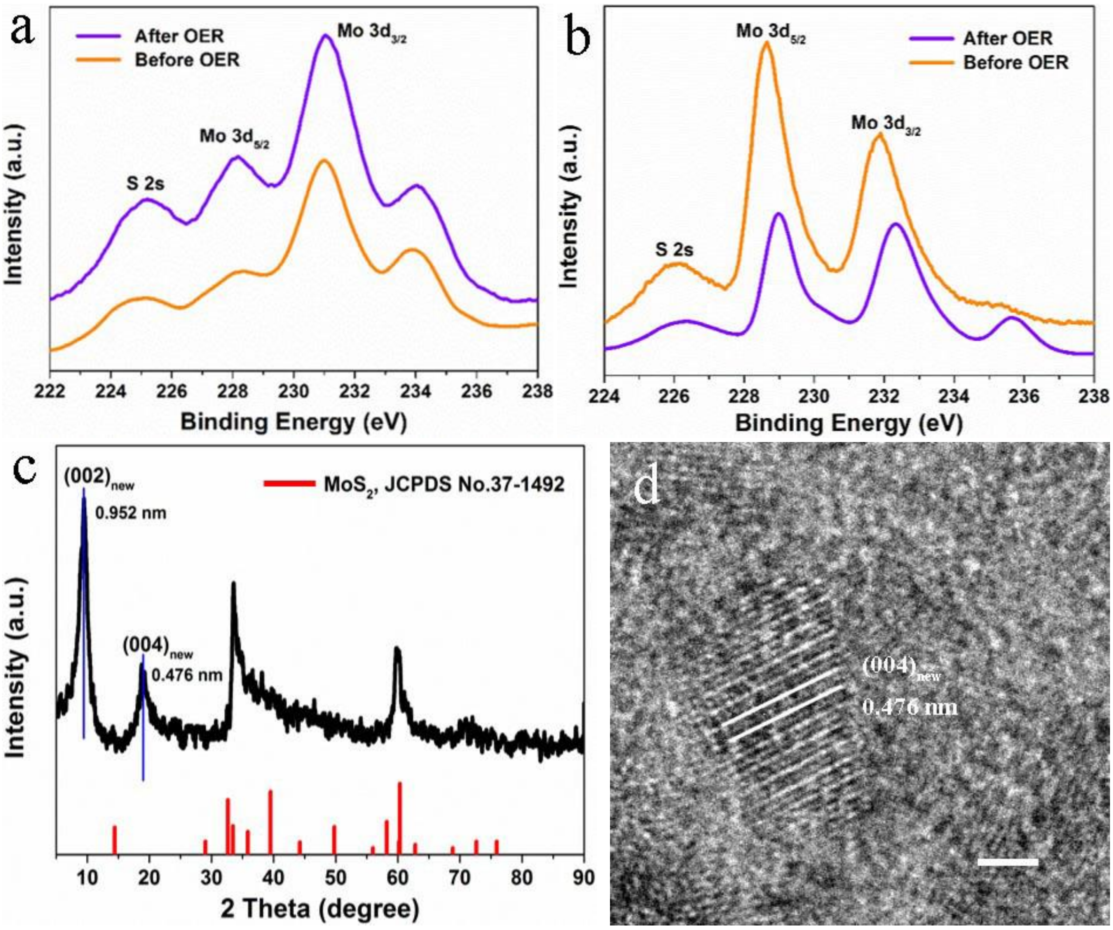


**Supplementary Figure 26.** XPS spectra of Mo in (a) 2.5H-PHNCMs and (b) 2.5H-$MoS_2$ before and after 1000 OER cycles. (c) XRD pattern and (d) HRTEM image of 2.5H-PHNCMs after 1000 OER cycles. Scale bar in (d), 2 nm.

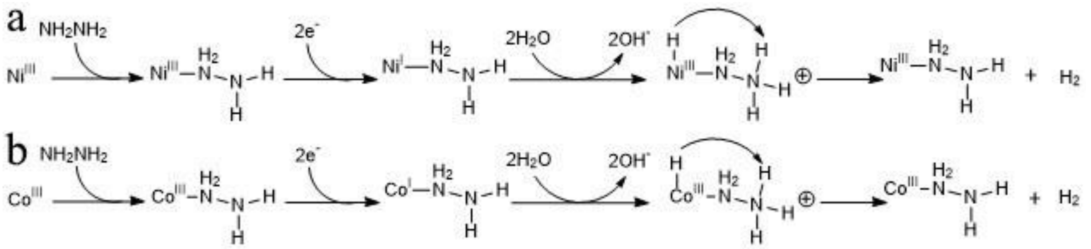


**Supplementary Figure 27.** (a) and (b) Possible mechanism of intramolecular proton transfer in hydrazine coordinated Ni and Co complexes.

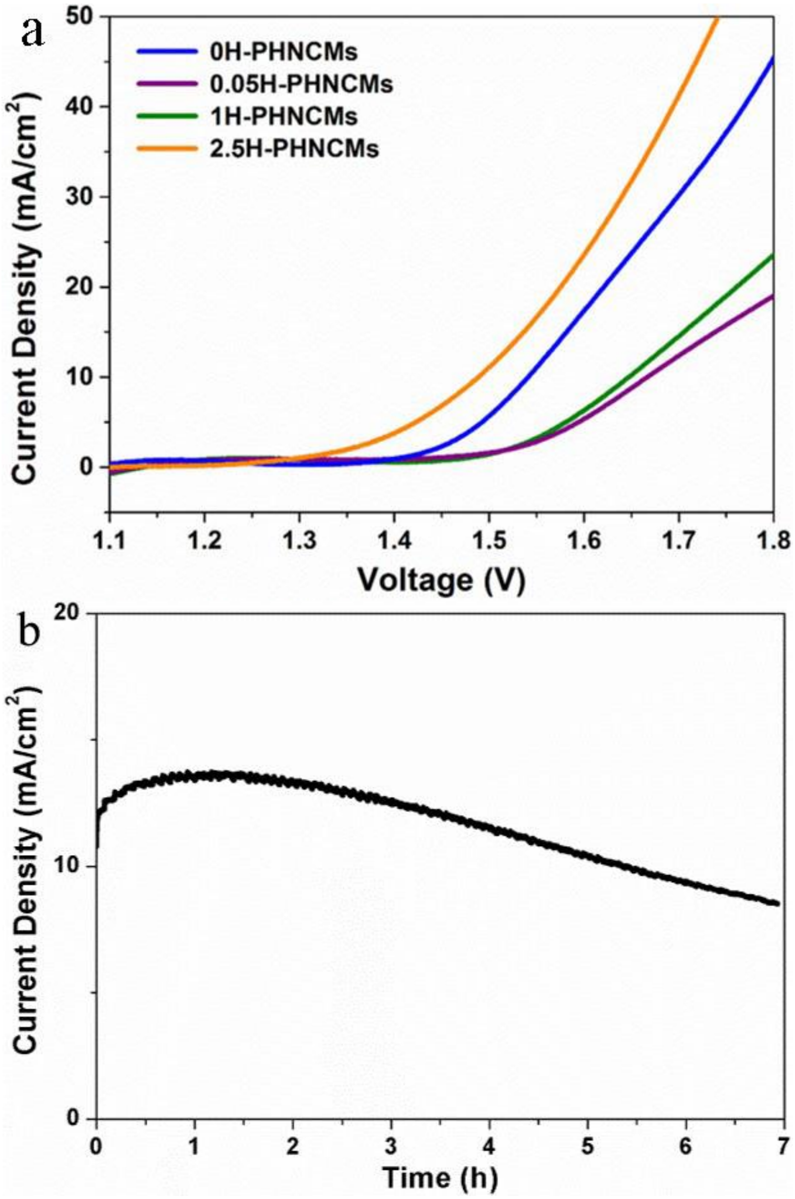


**Supplementary Figure 28.** (a) Steady-state polarization curves comparison for overall water splitting of PHNCMs at a scan rate of 5 mV·s$^{-1}$ in 1 M KOH electrolyte after a 20-minute open circuit scan. (b) chronoamperometric curve of overall water splitting on commercial $IrO_2$/C-Pt/C couple electrodes at a constant cell voltage of 1.65 V. The mass loading of each electrode is 1 mg·cm$^{-2}$.

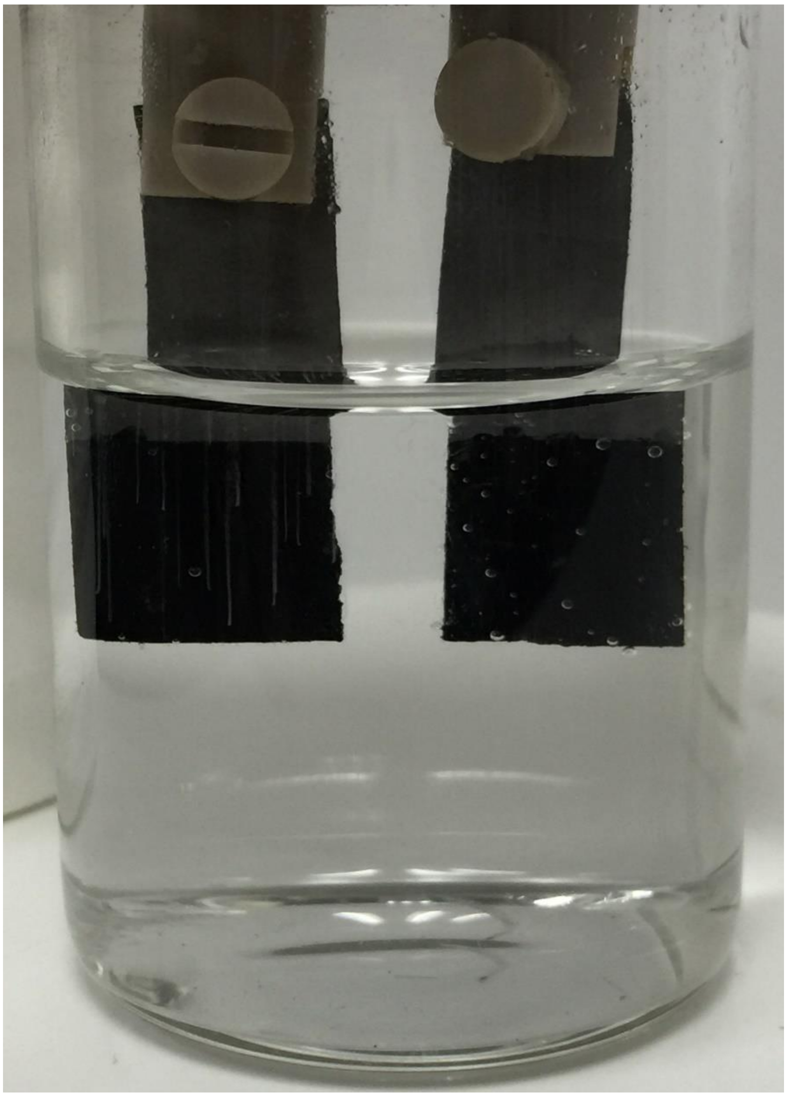

**Supplementary Figure 29.** A digital photo shows the $H_2$ (on the left electrode) and $O_2$ (on the right electrode) bubbles remained on the CFP daubed by 2.5H-PHNCMs after 24-hour chronoamperometric operation.

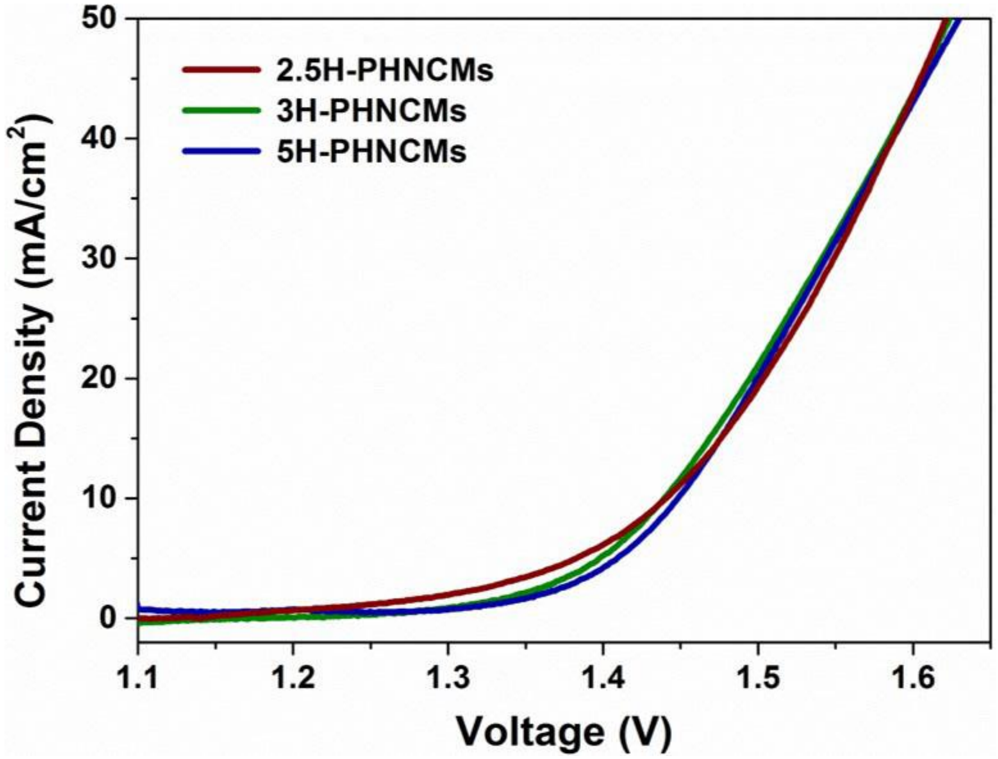


**Supplementary Figure 30.** Steady-state polarization curves of 2.5H-, 3H- and 5H-PHNCMs on CFP with 3 mg·cm$^{-2}$ of mass loading for overall water splitting in 1 M KOH electrolyte at a scan rate of 5mV·s$^{-1}$ with a two-electrode configuration after an open circuit scan for 20 min.
